# Addressing gender disparities in academic hospital medicine: The role of micro‐recognitions

**DOI:** 10.1002/jhm.70131

**Published:** 2025-07-13

**Authors:** Kristina M. Krohn, Beth K. Thielen, Jessica Hane, Caitlin Bakker, Katelyn M. Tessier, Sophia Gladding, Elizabeth A. Rogers, Michael B. Pitt, Taj Mustapha

**Affiliations:** ^1^ Department of Medicine University of Minnesota Minneapolis Minnesota USA; ^2^ Department of Pediatrics University of Minnesota Minneapolis Minnesota USA; ^3^ University of Regina Regina Saskatchewan Canada; ^4^ Masonic Cancer Center, Biostatistics Core University of Minnesota Minneapolis Minnesota USA; ^5^ Fairview Health Services Minneapolis Minnesota USA

## Abstract

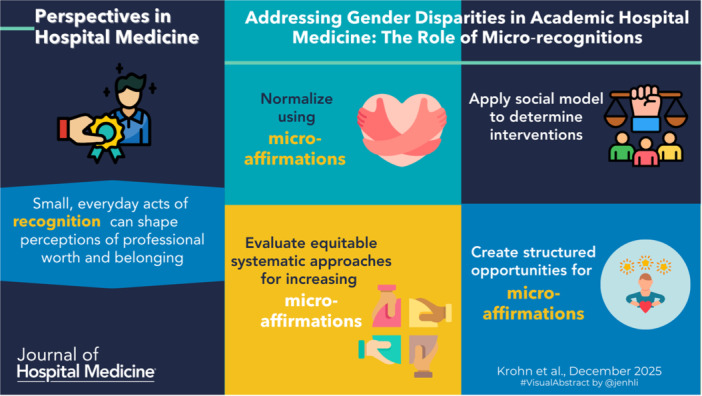

Diverse workforce and leadership teams in medicine are essential for achieving better patient care outcomes.^1^, ^2^ Previous research has shown that women physicians achieve lower mortality and readmission rates in their patients compared to their men peers.^3^ Other studies demonstrated that women adhere more to guidelines, provide better preventative care, offer more psychosocial counseling, spend more time with patients, and engage in greater patient‐centered communication.^3^ Despite achieving gender parity in medical school matriculation for over two decades, significant gender disparities persist in academic medicine. For academic hospital medicine, while there are equal numbers of men and women faculty, more men are division leaders.^4^ Gender disparities in academic rank across medical specialties are increasing, suggesting that structural challenges in faculty recruitment, retention, and promotion remain unaddressed.^5^ Prior efforts to mitigate these disparities, such as improving transparency, tracking gender‐specific data, enhancing mentorship, and promoting anti‐sexism training, have made some headway. However, further innovations are necessary. We advocate for adding a new approach: the identification, creation, and evaluation of “micro‐recognitions.”

## THE NATURE OF INEQUITIES IN ACADEMIC MEDICINE

Gender disparities in academic hospital medicine are often rooted in both macro‐ and micro‐inequities. Macro‐inequities, such as biased promotion systems, often reflect broader structural inequalities. To address these, large‐scale interventions like redistributing resources and holding leaders accountable for gender disparities are required. However, micro‐inequities—subtle, often unintentional actions like overlooking women's titles or failing to acknowledge their contributions—also play a significant role in perpetuating gender bias.^6^ Addressing these micro‐inequities can be challenging, but one method is improving micro‐affirmations.

Micro‐affirmations, small acts that convey respect and dignity, have been identified as essential for combating micro‐inequities.^5^ These subtle gestures may improve the sense of belonging and value for underrepresented individuals. For academic hospital medicine, micro‐recognition, a specific form of micro‐affirmation, may be effective due to the emphasis put on recognition in academia. The business world already invests in micro‐recognitions, due to evidence that improving micro‐recognitions improves employee retention and productivity.^7^ Although macro‐recognitions (e.g., promotions, tenure, grants) are essential for academic advancement, micro‐recognitions—small, informal acts that acknowledge faculty members’ work—may be equally important in building recognition and fostering an inclusive culture.

Here we propose a framework for increasing gender equity in academic hospital medicine through the enhancement of “micro‐recognitions”. The aim is to provide consistent, equitable recognition for the contributions of women, helping to address disparities in their career progression.

## THE ROLE OF MICRO‐RECOGNITION IN ACADEMIC ADVANCEMENT

For faculty members to be promoted or receive tenure, they must demonstrate regional, national, or international recognition of their expertise. Macro‐recognitions, such as prestigious publications, speaking engagements, and grant funding, are typically listed on a curriculum vitae (CV) and serve as formal indicators of a faculty member's accomplishments. However, promotion is also influenced by subjective factors, including the degree to which an individual's work is recognized and discussed within the academic community.^8^

Micro‐recognitions, while not always documented in a CV, can contribute significantly to a faculty member's reputation and career trajectory. For example, informal recognition through ad hoc emails acknowledging a colleague's publication or contributions can reinforce a sense of value, thus bolstering the individual's self‐efficacy. These small, everyday acts of recognition may play a crucial role in shaping faculty members’ perceptions of their professional worth and may serve as powerful tools for advancing gender equity in academic hospital medicine.

While men often self‐promote and self‐cite to gain micro‐recognitions, women are less likely to engage in self‐promotion—even when it would clearly benefit their advancement.^9^, ^10^, ^11^ When attributes deemed needed to be a leader are at odds with attributes considered feminine, role‐incongruity may occur leading to women leaders being penalized for acting against societal expectations.^11^, ^12^ Settings with a culture of frequent micro‐affirmations increased feelings of belonging and value for women and other marginalized groups, while targeted micro‐affirmations increased retention and success for underrepresented groups in STEM education.^13^, ^14^

As women report feeling less belonging and less congruence between their own values and those of their institutions throughout academic medicine, improving micro‐affirmations and micro‐recognitions may improve retention and promotion.

## PRACTICAL STRATEGIES FOR ENHANCING MICRO‐RECOGNITION

1. **Promote the use of micro‐affirmations**: Faculty members and leaders should understand the potential of micro‐affirmations and incorporate them into daily practices. The use of micro‐recognitions can be normalized within a hospital group or division. For example, a leader can make it standard practice to evaluate who talks at meetings. Then leaders can ask colleagues who are less likely to speak to share their expertise and pair this with a comment on their specific expertise. This both acknowledges their expertise and shows inclusion for someone who may feel undervalued. Further examples of micro‐recognitions are described in Table 1.
2. **Recognize the impact of recognition**: Micro‐recognitions can play a pivotal role in how an individual is perceived by their peers and superiors. Public recognition of faculty accomplishments can significantly enhance an individual's visibility and reputation. These informal recognitions contribute to the overall recognition needed for career progression.
3. **Create structured opportunities for micro‐recognitions**: The ad hoc nature of many micro‐recognitions can be affected by bias and role‐congruity/role‐incongruity. The effects of bias may explain why women generally receive fewer micro‐recognitions in academic medicine.^9^, ^15^, ^16^, ^17^ For example, email “shout‐outs” for publications in an internal medicine residency program were given more frequently to men than women, despite equal publication rates when done in an ad hoc manner, as the men tended to self‐promote.^9^ A systematic collection of publications from all the residents equalized the congratulatory emails.^9^
  At the University of Minnesota Pediatrics Department, we created two systems that provide micro‐recognition of scholarship (1) a newsletter where administrative assistants in each division submit the faculty members’ citations and (2) the Positive Peer‐Pressured Productivity (P‐QUAD) website. P‐QUAD is a platform developed by one of the authors that encourages faculty to log their publications and other scholarly activities in real‐time to be eligible for prizes. P‐QUAD provides a transparent and publicly visible record of self‐reported faculty productivity.^18^, ^19^ There was no difference in recognition in proportion to the number of publications by gender for both the departmental newsletter and P‐QUAD, revealing an equitable distribution of micro‐recognitions by gender. This suggests that systematizing micro‐recognitions can help close the gender gap in recognition.
4. **Apply a social model to determine micro‐recognition interventions**: The social model focuses on addressing how society and systems exclude or discriminate against a group of people. Adapting this model to the application of micro‐recognitions may identify areas for improving the system, as opposed to focusing on the individual.^20^ Instead of asking why women may not progress at the same rate as men, leaders should focus on what aspects of the system are preventing women from advancing. This lens encourages institutions to examine and address the biases embedded in the structures that may inadvertently hinder women's progress, such as relying on self‐promotion to acquire micro‐ and macro‐recognitions. From the above examples, the residency faculty did not blame men for self‐promoting or women for not self‐promoting but used the social model to change the system by having a clear expectation that everyone turn in scholarship activities as well as having administrative staff collect and track publications.
5. **Evaluate equity of systematic approaches for increasing micro‐recognition:** Leaders must regularly assess recognition practices for equity and actively seek out hidden opportunities for micro‐recognition within their departments. This includes tracking who receives praise or micro‐recognition for their work in newsletters, emails, and meetings. Part of creating equitable systematic approaches for micro‐recognitions may require evaluating institutional structures like mentorship programs, service assignments, and education roles. These positions require expertise but often receive less recognition. Assessing for equity in who receives micro‐recognitions allows institutions to identify when women or other underrepresented groups may be receiving less recognition for similar work or if the work tends to be allocated in gendered ways, as often occurs for service and education roles.
6. **Foster continuous improvement**: Medicine should not wait for another generation to prove that current practices are insufficient. We should start now and utilize quality improvement plan‐do‐study‐act cycles to make a difference in gender equity today, and a bigger difference tomorrow. As part of this process, faculty and leaders must remain open to feedback and willing to adjust to create a more inclusive and supportive environment for all.

**Table 1:** Examples of micro‐recognitions relevant for academic hospitalists by setting.

*Newsletter* a
Include a designated section with a list of all publications by hospitalists.^b^
Include a designated section with all recently completed projects (QI, mentoring, etc.).
Include a designated section with volunteer services to the group, community, hospital, etc.
Include a designated section to highlight peer and patient comments.
Include a designated space for kudos that may not fit into other sections.
*Division meetings* a
Track who speaks at meetings and reach out to those who speak less to ensure they can contribute.
Ask those who speak less if they have a preferred way to share their expertise.
When asking for a faculty member's input, note that it is valued due to their specific expertise.
Have the leader of the meeting acknowledge key accomplishments of faculty members.
*Grand rounds/presentations*
Ensure that proper titles are used during introductions and through question‐and‐answer sessions for all speakers/questioners.
Present colleagues as experts in their chosen fields during lectures and/or Q&A sessions.
When calling on a colleague for comment, state why their expertise adds to the conversation.
Provide thank you notes to all presenters or lecturers and consider cc'ing their department chair/supervisor.
*Hospital*
Acknowledging a colleague's recent accomplishment in the halls.
Thank a colleague for how their work made yours easier.
Thank a colleague for a quality sign out.
In notes, recognize the quality care done by a colleague, e.g. “I appreciate the thorough work up done by Dr. X during the last admission.” Or “I appreciate the important care conference held by Dr. Y on [date].”
Publicly acknowledge a colleague in writing (in addition to a verbal thank you) when they help cover a shift, take extra cross cover, take extra time with a family member, or take on extra time with learners.
*Any time*
Dual promotion—when sharing an accomplishment, mention others whose expertise assisted with the accomplishment.
Establish policy on when formal titles will or will not be used.

^a^ Newsletters and division meetings are listed first as they can be used to establish norms, expectations, and may be easiest to systematize and monitor.

^b^ Establishing expectations that all publications or projects are reported to the appropriate person to share in the newsletters is paramount for success of these measures.

## CONCLUSION

Addressing gender disparities in medicine requires both macro‐level interventions and attention to micro‐level inequities. While addressing the macro‐level disparities can seem overwhelming, improving micro‐recognitions is a concrete and achievable action at any level of hospital medicine, and by anyone—women and men allies alike. By enhancing micro‐recognition practices—such as establishing a culture of frequent small acts of acknowledgment of a colleague's work—institutions may be able to create a culture where all faculty feel valued and supported in their medical careers.

## CONFLICT OF INTEREST STATEMENT

The authors declare no conflicts of interest.

## ETHICS STATEMENT

This study was granted exemption status from the University of Minnesota Institutional Review Board on 3/12/2019 (ID 00005878).
